# The Role of Lateral Pelvic Lymph Node Dissection in Middle and Lower Rectal Cancer (Stage II or III): A Literature Review

**DOI:** 10.7759/cureus.67526

**Published:** 2024-08-22

**Authors:** Alexandra-Eleftheria Menni, Georgios Tzikos, Patroklos Goulas, Stylianos Apostolidis

**Affiliations:** 1 Department of Surgery, Aristotle University of Thessaloniki, AHEPA University Hospital, Thessaloniki, GRC; 2 First Propaedeutic Department of Surgery, Aristotle University of Thessaloniki, AHEPA University Hospital, Thessaloniki, GRC

**Keywords:** rectal cancer, review of literature, neoadjuvant chemoradiotherapy, lateral pelvic wall lymph node dissection, pelvic lymph nodes

## Abstract

Lateral lymph node dissection and its inclusion in the treatment of rectal cancer is a controversial issue, with great differences, especially between Eastern and Western countries. Studies try to highlight the superiority of resection of these lymph nodes compared to simple mesorectal resection in terms of local recurrence of the disease, the overall survival of patients, and additional postoperative complications. In this study, the modern literature was reviewed, with the ultimate goal of clarifying the exact importance of lateral lymph node dissection, in terms of oncological outcome in patients with cancer of the middle and lower rectum, by studying the involvement of this lymph node dispersion in terms of local recurrence and overall survival of patients with rectal cancer. This review was carried out using electronic databases, including PubMed, Embase, and MEDLINE, with studies dating back to the last decade. Of the 31 studies that were eventually included in the final review, there is no statistically clear superiority and real benefit from lymph node resection beyond the lymph nodes of the mid-rectum. European guidelines are set against lateral lymph node dissection, except for lymph nodes that show suspicious features on preoperative imaging. In contrast, in Eastern countries, total mesorectal excision (TME) with extensive simultaneous resection of the lateral pelvic lymph nodes (LPLNs) is the protocol followed. Recent studies focus on the subcategory of patients with non-responsive to adjuvant therapy, lateral lymph nodes, in which the ultimate benefit of extensive lymph node dissection is explored. The decision to join the TME procedure for the removal of the LPLNs is a subject of intense research. There are no data on the criteria for determining these lymph nodes as an increased risk of metastatic outbreaks. Despite the great clinical and research interest worldwide nowadays, the resection of LPLNs remains a controversial issue of debate, with intense disagreements according to geographical area, while the existence of additional studies is necessary to come to final conclusions.

## Introduction and background

Colorectal cancer is the most common gastrointestinal tract cancer for both men and women, with an increasing incidence in younger age groups [1]. Metastases to the lateral pelvic lymph nodes (LPLNs) are present in about 1/5 of patients with rectal cancer. In the majority of patients with lymph nodes suspected of metastatic disease on initial imaging, the presence of positive LPLNs persists after neoadjuvant therapy and surgery. The standard treatment includes neoadjuvant chemoradiotherapy, followed by surgery [2].

European guidelines generally tend to be against lateral lymphadenectomy. In contrast, in Japan, the protocol largely followed is total mesorectal resection with extensive simultaneous resection of the LPLNs [3]. It has been shown that the exact anatomical location of the tumor and the specific characteristics of the lymph nodes (size, capsule contour, and signal heterogeneity on imaging) play a decisive role in the correct choice of treatment [4].

This study is the result of a literature review of the most up-to-date data, analyzing the importance and ultimate benefit of resection of the LPLNs in rectal cancer. The main objective of this study is to clarify the exact significance of lateral lymph node clearance, in terms of oncological outcomes, for patients with middle and lower rectal cancer. This paper explores the data in the current literature, regarding the existence of a real benefit of more extensive LPLN dissection, in terms of recurrence and patients’ survival.

## Review

Materials and methods

**Figure 1:**
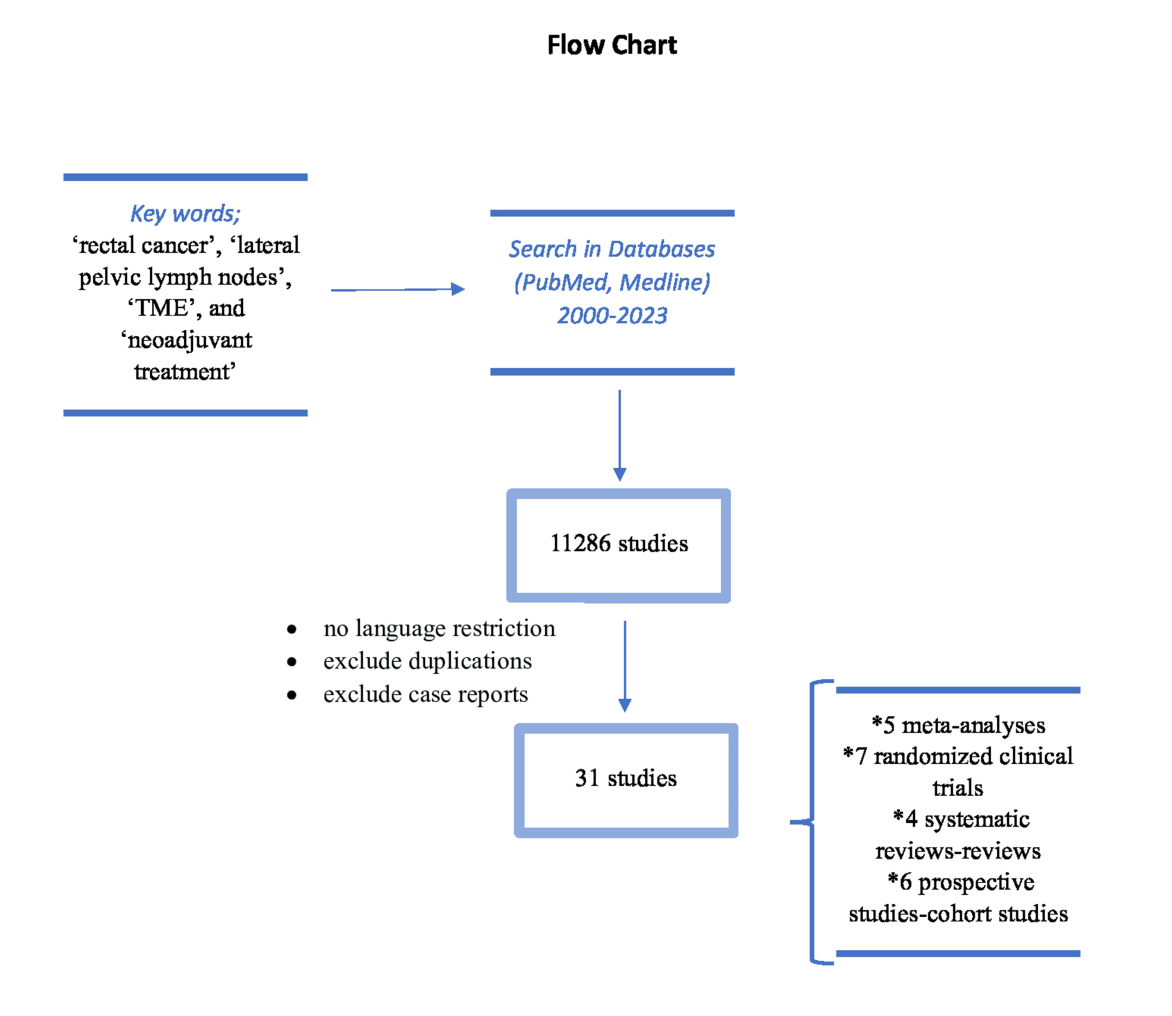
Flowchart

This study is the result of a literature review of the most up-to-date data, analyzing the importance and ultimate benefit of LPLN dissection. The data used were searched in scientific medical articles on the internet and in electronic databases such as PubMed and MEDLINE. During the search of the studies, keywords, including "rectal cancer," "lateral pelvic lymph nodes," "TME," and "neoadjuvant treatment," in free text format, as well as classification terms of the bibliographic databases were used. This literature review yielded a total of 11286 studies, chronologically embedded between 2000 and 2023, according to specific combinations of search words. The review of the literature was conducted by two independent authors. The articles were collected based on the keywords, after removing duplicates, and after consensus for articles that they did not agree to. There is no language restriction in the search, nor strict criteria in terms of patient selection. The final selection was done by another third researcher. Finally, a total of 31 studies were included in the study, of which five were meta-analyses, seven were randomized clinical trials, four were systematic reviews and reviews, and six were prospective studies and cohort studies (Figure 1).

Literature review - discussion

The latest treatment approach for locally advanced rectal cancer is a combination of various methods. In North America and Europe, complete resection of the tumor and the rectum, together with all possible organs to which it extends, combined with neoadjuvant chemoradiotherapy (CRT) is the treatment of choice [5]. However, as found in Eastern studies, in 10-25% of cases, there are positive lymph nodes, which, being outside the mesorectum, are not included in the initial histological smear [6]. The rectum is drained through two main routes: from the inferior mesenteric artery in the upper third to the para-aortic lymph nodes, and from the middle and inferior rectal artery to the internal iliac artery, to the obturator, medial, and external iliac lymph nodes. According to the protocol of classical total mesorectal excision (TME), the vast majority of lymph nodes in the first route will be excluded, unlike the lymph nodes described in the second category. The main geographic variation in patient selection for treatment lies in the fact that positive internal iliac lymph nodes are considered a local recurrence of the disease in the West, whereas any positive external and common iliac lymph nodes are considered distant metastases [7]. In contrast, according to the Japanese guidelines, all pelvic lymph node metastases are considered to be locoregional.

Canessa et al. divided the pelvic lymph nodes into three main subgroups: pre-pelvic lymph nodes, hypogastrium nodes, and around the obturator nerve ones. This study showed that the main presence of positive lymph nodes was found along the internal pudendal artery, the internal iliac artery, and the region of the psoas muscle and obturator nerve, classifying these areas as "vulnerable fields." The increased incidence of metastases in these areas is justified by the lymphatic drainage from the lower rectum, into the mediastinum and then through the lateral ligament, along the internal iliac artery to the area around the obturator nerve. It is worth emphasizing that the three areas mentioned are oncologically related because of their direct relationship to the peripheral border of the tumor [8].

Positive LPLNs are associated with poor prognosis, and a reduction in five-year survival to 25%, down from 74% for patients with negative lymph nodes. Although treatment remains equivocal, defining the risk factors for having positive LPLNs is required to target patients for LPLN clearance. Female gender, moderate/low differentiation tumors, lymph nodes (LN) >4 mm in size, T3-T4 stage disease, and tumors with reduced distance from the anal canal are considered to be at increased risk for the presence of positive LPLN. In addition, lymph nodes suspected of malignancy are judged according to their size (>5/7/8 mm in maximum and minimum diameter) and also based on their specific characteristics as well as their borders. In terms of the size of the lymph nodes in relation to the likelihood of their malignant outgrowth, the use of different size criteria (cut-off values) has led to enhancements in the accuracy of preoperative diagnosis for LPLNs; nevertheless, employing the common criteria of a short axis ≥4 mm and a long axis ≥7 mm did not result in a substantial improvement in diagnosis accuracy beyond 80%, despite variations in sensitivity and specificity. To conclude, the size alone should not be a criterion for the evaluation of metastatic disease, especially for intermediate lymph node sizes, 4-8 mm, which remain particularly controversial and complicate the therapeutic choice and oncological evaluation of patients. Nevertheless, the circumferential tumor margin (CRM) remains the main prognostic factor for local disease control in locally advanced rectal cancer [9].

Several clinical studies have described the therapeutic benefits of neoadjuvant therapy in terms of local disease control. However, when data from the Dutch clinical trial, were compared with similar results from the National Cancer Center in Japan, no statistically significant differences were observed between TME resection combined with preoperative radiotherapy and TME together with lymph node resection. Due to the variety of outcomes, the need for lymph node dissection in patients undergoing neoadjuvant radiotherapy, the benefit of which is generally proven, remains uncertain but is not routinely used in all colorectal cancers due to increased toxicity and associated complications.

Studies from Japan comparing preoperative CRT with or without concomitant lymph node clearance concluded that LPLN resection is not necessary as a therapeutic modality in patients with advanced lower rectal cancer and that neoadjuvant radiotherapy may be an alternative therapeutic strategy to lateral lymph node clearance [10].

Despite the amount of studies, and the variety of results, especially between Western and Eastern countries, the selection of patients who will have a proven benefit from lymph node dissection remains crucial. Even more, a positive prognostic factor is the response of the lymph nodes to CRT. Kim et al. demonstrated that non-suspected lymph nodes and lymph nodes with good response to neoadjuvant therapy have similar outcomes in terms of relapse-free survival and overall patient survival. In this review, the need to find a single strategy as the treatment of choice is emphasized, suggesting the following treatment options: in low-risk patients, classical TME resection is sufficient. For moderate-risk patients, neoadjuvant CRT combined with TME resection of the tumor or TME resection with concomitant lateral lymph node clearance, and finally, for higher-risk patients, neoadjuvant treatment followed by TME resection with concomitant lateral lymph node cleansing is considered beneficial, especially in cases of non-response of the lymph nodes to preoperative CRT [11].

In 2019, Atef et al. conducted a systematic review that included patients with confirmed lymph node metastases on preoperative imaging who underwent TME resection with concomitant lymph node clearance after neoadjuvant radiotherapy [12]. This review is the first study with confirmed pathological lymph nodes under neoadjuvant CRT (preoperative chemo-radiotherapy). Even patients considered to have had a complete response to preoperative treatment were found to have positive lymph nodes at an increased rate. This led to the conclusion that lymph node resection has an unquestionable place in all cases of non-responsive lymph nodes, but even for those who respond to radiotherapy, it could show some benefit. In particular, patients with lymph nodes that responded to neoadjuvant therapy have a 0-20% chance of positive metastases, while for those who did not respond, the rate is 25-83%. Total mesorectal resection with or without neoadjuvant therapy as well as TME with concomitant lymph node cleansing without radiotherapy proved to be a method of treatment approach for rectal cancer, showing good local disease control compared to TME resection alone. In addition, analysis by Kim et al., studying long-term outcomes, demonstrated that patients with lymph nodes responsive to radiotherapy may not show benefit from lateral lymph node cleansing in terms of local recurrence and overall disease survival, in contrast to patients with lymph nodes resistant to neoadjuvant therapy, who benefit from their subsequent resection [11].

In a 2018 review, Sammour et al. compared the results between lateral lymph node clearance with simple TME resection in patients with negative lymph nodes on preoperative imaging and demonstrated that local recurrence is reduced with resection of abnormal lymph nodes and therefore the superiority of TME was not proven. Therefore, lateral lymph node clearance may reduce local recurrence in the absence of neoadjuvant therapy. The diversity of patients, in terms of stage of disease, suppressed or non-suppressed lymph nodes, their size, and the general characteristics of the disease in each patient, also require individualization in the treatment of choice, despite the attempt to classify patients into three categories, according to risk [13].

A randomized study, originally published in 2017 (JCOG0212), enrolled patients from 2010 to 2017, with stage II-III rectal cancer, localized below the peritoneal recurrence, with concomitant lymph nodes <10 mm in size. Patients were divided intraoperatively, randomly into TME simple resection and TME with concomitant lymph node clearance. The patients were followed up every four and six months, with particular focus on three sites of metastases location: central pelvis, lateral pelvis, and anastomosis area [14]. The primary end-point was defined as relapse-free survival, for which no significant difference was observed between the two subgroups of patients (73.4%-73.3%). The secondary catalytic point was defined as overall survival (92.6%-90%), local recurrence (87.7%-82%), incidental events, blood loss, sexual dysfunction, and urological disorders. The only statistically significant difference observed was a reduction in recurrence in the lateral pelvic wall in the subgroup with subsequent lymph node dissection. Because of this, lateral lymph node clearance was recommended for patients with confirmed lymph node metastases on imaging, to the extent that staging is possible preoperatively. Of particular importance is the careful selection of patients as lymph node dissection is associated with increased operative time and increased blood loss intraoperatively. At the time of this protocol, neoadjuvant radiotherapy had not been included in the treatment routine of patients as there were no clear results regarding its ultimate benefit and safety, resulting in its generalized use in Japan. The recurrence rate nevertheless remained at 10%, with 14 patients presenting with micro- and macro-residual disease. This prompted the inclusion of neoadjuvant therapy in the treatment of choice for these patients, as it was found that surgery alone could not guarantee the eradication of the disease [14]. Initially, the study aimed for a five-year follow-up of patients; however, from the limitations and results obtained during this period, it was decided to extend the follow-up of patients to seven years. The new results of the two subgroups of patients (TME and TME with lymph node clearance) were as follows: in terms of overall survival, 86.8% for the subgroup with concomitant lymph node clearance, versus 84% for the subgroup with TME resection. Concerning local recurrence, the rates ranged from 7.5% for patients with lymph node resection while for those who had a simple TME, the rate was 12.7%. Finally, as for the disease-free survival (recurrence-free survival), the rates were 82.9% for patients who underwent lymph node clearance, and 78.9% for the subgroup with conventional TME resection. In addition, patients with lymph node size less than 7 mm had local recurrence-free rates of 85.1%, and with lymph nodes between 7-10 mm in diameter, the rate was 70% [15].

In particular, recurrence-free survival (RFS) showed better rates in patients who also underwent further lymph node resection. However, no statistically significant difference was observed between the two subgroups of patients for those with stage II cancer. Among patients with stage III cancer, no statistically significant rates of better response were observed in patients who underwent lymph node resection. Finally, no difference in RFS was observed comparatively between stage I and II patients.

RFS at seven-year follow-up of patients was above 70% for both categories of patients. The overall five-year survival for patients with stage II-III cancer, who underwent neoadjuvant therapy, followed by TME with conjoint lateral lymph node clearance ranged between 59% and 88%. In this study, the five-year survival of patients who underwent simple TME resection and TME with lymph node clearance was 90.2% and 92.6%, respectively. The increased oncological outcome, therefore, once again leads to the conclusion that the non-superiority of classical TME resection compared to TME and lymph node clearance cannot be proven [15].

In addition, Sapci et al., in 2018/2019, conducted an observational study in which they compared patients with stage III rectal cancer, who were divided according to the findings of preoperative MRI into those who had suspicious lymph nodes before neoadjuvant treatment and those who did not have lymph nodes suspicious for malignancy features observed on imaging. The results showed that local recurrence was increased in the subgroup of patients with suspicious lymph nodes, but without demonstrating a statistically significant difference. Furthermore, a decrease in five-year survival was observed in patients with radiologically suspicious lymph nodes, which showed a tendency to improve with the application of preoperative radiotherapy. Nevertheless, the study concluded that the presence of suspicious LPLNs affects local recurrence even in those cases where a response of these lymph nodes to neoadjuvant therapy is observed. Notably, patients with good radiotherapy response of their lymph nodes had worse rates of local recurrence compared to those patients who did not present suspicious pelvic lymph nodes at preoperative staging [16].

Based on this conclusion, Sugihara et al. argued that lateral lymph node clearance can reduce pelvic recurrence by up to 50%. As a result of all this, and given a recent randomized controlled trial that showed that the combination of TME with additional lateral lymph node dissection can lead to a reduction in local recurrence, more recent data concluded that resection of LPLNs should perhaps be performed in the case of imaging evidence of lymph nodes suspected of malignancy [17]. However, the evidence remains incomplete and does not allow a definitive conclusion to be drawn [16,17].

A recent study divided patients into two groups according to whether they had lymph node metastases in the MRI before radiotherapy. Of the 20 patients with positive lymph nodes, 15.8% had recurrence (p = 0.039) and disease-free survival was 56.2%. The corresponding rates for patients without positive lymph nodes before neoadjuvant therapy were 2.3% and 87.3%. In conclusion, LPLNs positive for metastasis cause higher local recurrence rates after neoadjuvant CRT and TME resection, and therefore, lateral lymph node dissection should be considered in the treatment plan for rectal cancer [18].

A meta-analysis conducted in 2019 in China analyzed and compared the benefits and potential drawbacks between two subgroups of patients with different mildly therapeutic approaches. More specifically, patients were classified into two categories: those who underwent preoperative radiotherapy with subsequent TME resection (CRT + TME), and those who underwent neoadjuvant therapy and TME, and LPLN exclusion (CRT + TME + lateral lymph node dissection) in addition to neoadjuvant therapy and TME [18]. Results demonstrated a significant reduction in the risk of lateral-pelvic local recurrence in the sub-group of patients with lateral lymph node clearance, from 7.9% to 2.9% (p = 0.02), especially in those patients who were clinically suspected of lateral lymph node metastases preoperatively. However, local disease recurrence, overall patient survival, and disease-free survival did not differ between the two groups of patients. Regarding the secondary catalytic points of this meta-analysis, a difference was shown only about urological and sexual disorders postoperatively for patients who ultimately underwent lateral lymph node clearance, for whom the rate of potential complications was significantly increased. However, except for an increase in operative time, no differences were observed in terms of intraoperative blood loss, anastomotic leakage, and wound or pelvic perforation [19].

A subsequent meta-analysis in November 2020, having local recurrence as the primary catalyst, divided patients into two subgroups: 2000 patients who underwent simple TME resection, and 1563 subjects in whom additional lymph node clearance was performed. It turned out that in the first group of patients, local recurrence was marginally increased; however, this was not statistically significant (9.8% vs. 9.4%, p = 0.35). Regarding distant metastases, reduced rates were observed in the extended lymph node dissection group: 27.3% vs. 29.9% (p = 0.02). Therefore, this meta-analysis concluded that extended lymph node dissection, compared to standard TME, is not significantly superior in terms of the risk of local recurrence of the disease [20].

A review and meta-analysis conducted in December 2020 included 29 studies and a total of 10,646 patients and focused on recurrence rates, both topographical and distant sites, overall survival, and disease-free survival, as well as postoperative complications. Of all patients reported, 39% underwent TME with concomitant lymph node clearance. From the results, no differences were observed between the two subgroups of patients in terms of sexual postoperative dysfunction in males, disease recurrence, or overall survival and complication-free survival. The two patient groups differed in terms of overall complications (p = 0.01) and urological disorders (p = 0.008), with a predominance of extended lymph node dissection. In conclusion, once again, the superiority of lateral lymph node clearance oncologically was not proven, and the higher rates of postoperative complications associated with it were demonstrated [21].

A meta-analysis as to the superiority, or otherwise, of lateral lymph node clearance was published in March 2021. This included 21 studies, 19 non-randomized clinical trials, and two randomized trials, between 1989 and 2020, with the majority of studies coming from the East. The results were obtained by comparing 4361 patients who underwent lymph node clearance and 4034 patients in whom simple TME resection was followed. Furthermore, no significant differences were observed in terms of overall five-year survival, nor in terms of five-year disease-free survival. Local disease recurrence in the non-randomized studies was worse in patients undergoing lymph node clearance (RR = 1.41 (95% CI: 1.21-1.64), p < 0.001), as was overall disease recurrence (RR = 1.44 (95% CI: 1.25-1.67), p < 0.001), while no differences were observed to distant metastases. These results are inconsistent with non-randomized studies such as JCOG0212 [14]. However, due to the high heterogeneity in this meta-analysis, more data are needed to definitively produce meaningful results [22].

Finally, the most recent meta-analysis in 2022, compared the results in patients diagnosed with lymph node metastases, either following TME resection and lymph node cleansing, or TME alone, after neoadjuvant therapy. In total, seven studies with a total of 946 patients were included in this meta-analysis. It was shown that local recurrence was clearly reduced in the group of patients following lymph node clearance, at 3-15%, compared to 11-27% in the group undergoing TME resection alone (p < 0.0001). However, in terms of five-year disease-free survival and five-year overall survival, no significant difference was observed between the two subgroups of patients. Therefore, it was concluded that LPLN dissection reduces local disease recurrence but is not associated with patients’ survival [23].

To sum up, we can conclude that the combination of TME with preoperative and/or adjuvant radiotherapy is the routine treatment for the management of patients with mid and lower rectal cancer in countries in the Western world. In the East, and particularly in Japan, the guidelines include in the protocol for the treatment of rectal cancer the extension of surgical resection beyond the limits of classical TME to the removal of LPLNs. The existence of metastases to lymph nodes outside the rectum and surrounding adipose tissue has been shown to be responsible for local recurrence rates [24]. A variety of studies have attempted to demonstrate the superiority of extended surgery, either in comparison with radiotherapy or in improving oncological outcomes in combination with it. Despite encouraging results in overall patient survival, the superiority of more extensive surgery has not been statistically proven.

## Conclusions

Total resection of the mesorectum is the gold standard for the radical and effective treatment of rectal cancer. In general, in low-risk patients, TME resection alone is sufficient. East and West persist in their differences in the treatment of rectal cancer, making it impossible so far to finalize a single treatment of choice. In conclusion, the literature to date remains limited, and new and diverse studies are needed on this highly controversial topic to derive the definitive and most oncologically appropriate treatment of rectal cancer.
